# Multidisciplinary Management of Neuroendocrine Neoplasms Beyond Centers of Excellence: Retrospective Real-World Data from a Referral Center in Eastern Europe

**DOI:** 10.3390/jcm15197762

**Published:** 2026-10-08

**Authors:** Irina Sandra, Elena Gabriela Chiorean, Irina Mihaela Cazacu-Croitoru, Vlad Mihai Croitoru, Simona Olimpia Dima, Adrian Goldiș, Carmen Monica Preda, Rucsandra-Ilinca Diculescu, Adina Emilia Croitoru, Mircea Mihai Diculescu

**Affiliations:** 1 Faculty of Medicine, “Carol Davila” University of Medicine and Pharmacy, 050474 Bucharest, Romania; 2 Department of Medicine, Section of Hematology-Oncology, University of Chicago, Chicago, IL 60637, USA; 3 Department of Oncology, Fundeni Clinical Institute, 022328 Bucharest, Romania; 4 Department of General Surgery, Fundeni Clinical Institute, 022328 Bucharest, Romania; 5 Faculty of Medicine, “Victor Babeș” University of Medicine and Pharmacy, 300041 Timisoara, Romania; 6 Department of Gastroenterology and Hepatology, Fundeni Clinical Institute, 022328 Bucharest, Romania

**Keywords:** neuroendocrine neoplasms, real-world data, multidisciplinary care, PRRT, liver-directed therapy, health-care access, Eastern Europe

## Abstract

**Background and Objectives:** Neuroendocrine neoplasms (NENs) require multidisciplinary management, yet access to advanced imaging and therapies is uneven across Europe. We aimed to describe real-world management of NENs in an Eastern Europe referral center. **Methods:** This retrospective single-center study included 192 NEN patients treated between 2011 and 2026 at a national referral center in Romania. **Results:** Most tumors were well-differentiated (88%), pancreatic primary (59.9%), and non-functioning (77.1%); 51.0% were metastatic at diagnosis. Overall, 30.7% (*n* = 59) of patients underwent somatostatin receptor imaging, mainly with locally available 99mTc-HYNIC-TOC scintigraphy (*n* = 56). Among therapeutic modalities, 68.2% of patients underwent conventional systemic therapy and 69.3% had surgery (R0 resection was achieved in 98.9% of curative-intent surgeries; recurrence rate 40%). Peptide receptor radionuclide therapy (PRRT) was performed in 14.6% (19/130) of metastatic NENs and liver-directed therapy (LDT) in 13.6% (16/118) of NENs with liver metastases. Median time to treatment was 78 days for somatostatin analogs, 29 days for chemotherapy in neuroendocrine carcinoma, 30 days for surgery, 62.5 days for LDT, and 144 days for PRRT. **Conclusions:** This is one of the few reports on real-world management of NENs in an Eastern European tertiary care referral center, which highlights the available multidisciplinary care but also the challenges in diagnostics and therapeutic access for patients with NEN.

## 1. Introduction

Neuroendocrine neoplasms (NENs) are a group of heterogeneous tumors with variable clinical behavior. Complex cases require a multimodal approach that includes surgery, systemic therapies, peptide receptor radionuclide therapy (PRRT), and locoregional procedures [1].

In routine practice, access to treatment and expertise is uneven across Europe. While dedicated Centers of Excellence and high-volume centers provide comprehensive care, access to advanced somatostatin receptor imaging or specialized therapies is not uniformly available in smaller hospitals [2,3]. For example, PRRT is unavailable in Romania, even in large tertiary care centers, and eligible patients must be referred abroad [4]. Furthermore, the expertise and infrastructure for selective internal radiation therapy (SIRT) have been available since 2025, but the procedure is not reimbursed by the national healthcare system. Across other Eastern European countries, access to specialized therapies and advanced procedures is variable, particularly in terms of availability and reimbursement [5]. In addition, formally accredited Centers of Excellence remain limited in the region [6]. Nevertheless, favorable outcomes can be achieved through appropriate management, including surgery, systemic therapies, liver-directed therapies, and appropriate follow-up [7].

Despite the growing incidence of NENs [8], data from Eastern Europe are heterogeneous and limited in several settings, with the available literature largely consisting of small retrospective cohorts and case series. To address this knowledge gap, we aimed to describe the real-world management of NEN patients in an Eastern European reference center for digestive oncology that is not formally accredited as a Center of Excellence by the European Neuroendocrine Tumor Society (ENETS). We focused on multidisciplinary treatment patterns and parameters of access to care, including time to treatment.

## 2. Materials and Methods

### 2.1. Data Collection and Variables

This retrospective study included patients with NENs from a single academic center, Fundeni Clinical Institute in Bucharest, Romania, which is a national referral institution for digestive NENs. Patients with a diagnosis of NEN who presented to our institution between January 2011 and January 2026 were identified through the electronic database implemented in 2011, and data extraction was initiated on 30 January 2026. No formal sample-size calculation was performed; all eligible patients during the study period were included. For 12 patients diagnosed before 2011 with incomplete data, archived paper records were additionally reviewed only for missing data, using the same definitions for the variables. Multidisciplinary tumor board (MDT) recommendations, if available, were also reviewed. Data on demographics, pathology, treatments, and outcomes were collected.

The present cohort partially overlaps with the pancreatic NEN cohort previously reported from our institution [7]. The previous study focused specifically on survival outcomes in surgical and non-surgical pancreatic NENs, whereas the present analysis includes NENs of all primary sites and focuses on multimodal management.

### 2.2. Variables and Statistical Analysis

Tumor stage was classified according to the 2017 TNM classification (8th edition) [9]. Tumor grade was reassessed retrospectively based on Ki-67 values according to the 2022 WHO classification [10]. Tumor response was assessed based on RECIST 1.1 criteria [11] by experienced radiologists at our institution. Postoperative complications were graded retrospectively according to the Clavien–Dindo classification: grades I–II were considered minor and III–V major. Disease-free interval (DFI) was defined as the interval from surgery to disease recurrence. Time-to-treatment intervals were assessed from first presentation to our center to first-line treatment initiation consisting of somatostatin analogs (SSAs), chemotherapy in neuroendocrine carcinomas (NEC), or surgery in our center (including primary tumor resection, cytoreductive surgery, metastasectomy, ablative procedures), as recommended according to National Comprehensive Cancer Network (NCCN) and European Society for Medical Oncology (ESMO) guidelines. The first presentation at our center was used as a reference point because the date of diagnosis was recorded heterogeneously (e.g., histopathological or imaging diagnosis). For liver-directed therapies (e.g., transarterial bland-, chemo-, or radio-embolization, radiofrequency ablation) and PRRT, which were mostly performed in later treatment lines, the interval was calculated from treatment recommendation to procedure, using the date of the multidisciplinary team (MDT) or specialist consultation.

Descriptive statistics were calculated using Microsoft Excel 2024 (Microsoft Corporation, Redmond, WA, USA). Continuous variables were reported as medians with interquartile ranges (IQRs), and categorical variables as frequencies and percentages. Time-to-event outcomes (overall survival [OS] and DFI) were estimated using the Kaplan–Meier method (Greenwood standard errors, log–log confidence intervals), and follow-up was estimated using the reverse Kaplan–Meier method; OS was calculated from the date of NEN diagnosis to death from any cause or to the censoring date of 30 January 2026 for patients alive. To explore residence and age as potential effect modifiers of treatment access, categorical associations, including exploratory analyses of PRRT and LDT by residence and age stratum (using an arbitrary cutoff of 65 years), were tested with the chi-squared test (Yates’ correction for 2 × 2 tables); estimates were not adjusted for other covariates, and no correction for multiple comparisons was applied. Sensitivity analyses for time-to-treatment metrics affected by exclusion or missing data used complete-case, pooled, and best-/worst-case imputation (0 days/95th percentile). Missing data for other variables were handled by complete-case analysis. All analyses other than descriptive statistics were performed in R version 4.3.2 (R Foundation for Statistical Computing, Vienna, Austria, survival package), following pre-analysis data quality checks.

The study was approved by the Ethics Committee of Fundeni Clinical Institute (Ord. MS 1502/2016, 223/20.04.2025) and conducted in accordance with the principles of the Declaration of Helsinki. Full access to all data in the database was granted indefinitely until the database was shut down. This application was approved on April 20, 2025, authorizing the authors to retrieve and analyze data between April 2025 and April 2027. The authors performed data retrieval and analysis in January 2026.

### 2.3. Eligibility Criteria

A confirmed diagnosis of NEN was mandatory. Documented primary tumor site (or confirmed unknown primary tumor), available tumor grade and stage at diagnosis, and at least one recorded treatment line in our center were also required.

Patients with adenocarcinoma, mixed neuroendocrine–non-neuroendocrine neoplasm, primary prostate NEN, medullary thyroid carcinoma, pheochromocytoma, or paraganglioma were excluded. Patients referred exclusively for consultation or second opinion without further management in our institution were also excluded.

## 3. Results

### 3.1. Patient Characteristics

A total of 253 NEN patients were identified, and 192 eligible patients were included in the analysis (patient selection flowchart, Supplementary Figure S1). The number of new case presentations increased considerably after 2016. Median follow-up was 64 months (CI 95% 58.7–79.8). At the time of analysis, 121 patients (63%) were alive. Vital status was known for the entire cohort; however, clinical follow-up data had not been updated for 27 patients (14.1%) lost to active follow-up, and the date of death was unavailable in two cases.

Median age at diagnosis was 60 years, and the distribution between sexes was balanced. The place of residence was equally distributed between the capital city, other urban areas, and rural areas. Most tumors (88%) were G1 or G2. In 10 cases, subsequent biopsies noted changes in Ki-67 index, resulting in reclassification to a higher grade. More than half of the tumors originated in the pancreas (60%), and the majority were non-functioning. Among functioning tumors, carcinoid syndrome was the most frequent (70.5%). Single cases of multiple endocrine neoplasia 1 (MEN 1) syndrome and von Hippel–Lindau (VHL) syndrome were reported. Twenty-seven patients had at least one other malignancy in addition to NEN. The majority had a good performance status at presentation (93.7% ECOG 0–1). Patients’ characteristics are detailed in Table 1.

**Figure 1:**
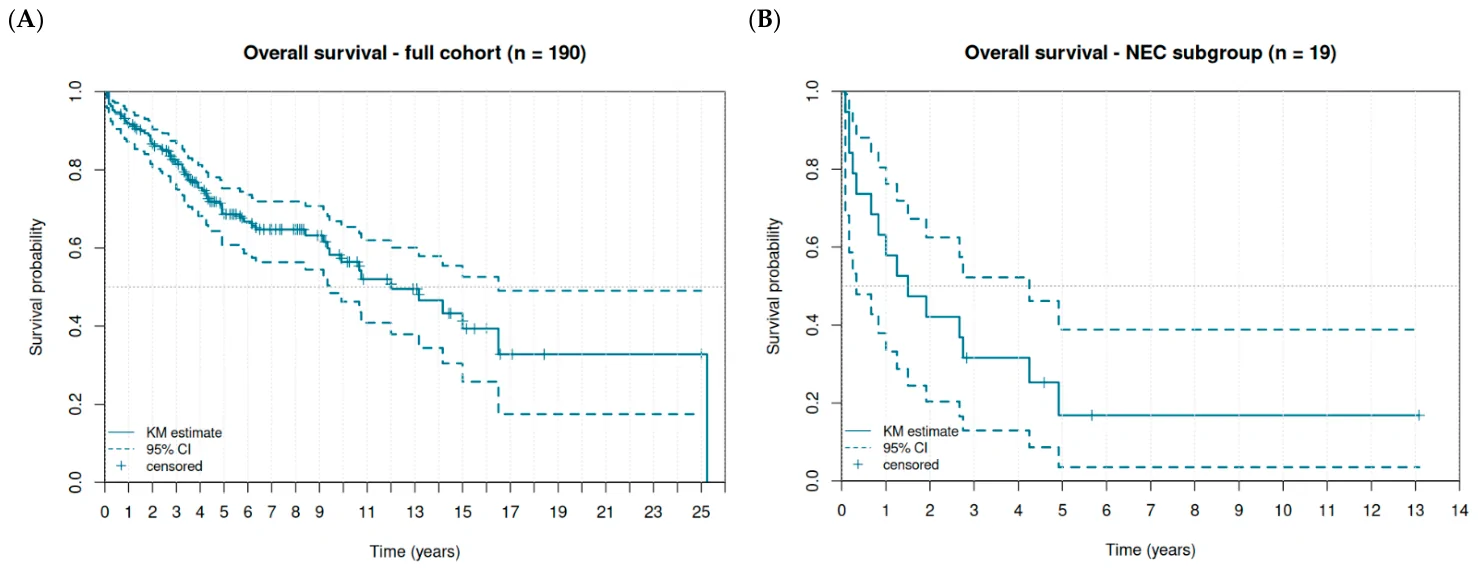
Kaplan–Meier overall survival curves for (**A**) the entire cohort (*n* = 190/192 with available data) and (**B**) patients with neuroendocrine carcinoma (NEC) (*n* = 19/19 with available data).

Median overall survival in the entire cohort was 144 months, with 5- and 10-year survival rates of 68.6% (95% CI, 60.8–75.3) and 56.4% (95% CI, 46.3–65.4), respectively. Patients with NEC had a median overall survival of 18 months, with a 1-year survival rate of 57.9% (95% CI, 33.2–76.3). The corresponding Kaplan–Meier survival curves are shown in Figure 1.

Half of the cohort (51%) had metastases at initial presentation, mostly involving up to three sites, and an additional 16.7% (*n* = 32) developed metachronous metastases during follow-up. The liver was the most common metastatic site (90.8%; 118/130), followed by lymph nodes (45.4%, 59/130), bones (23.8%, 31/130), peritoneum (23.1%, 30/130), and lungs (17.7%, 23/130). Among patients with liver metastases, 48.3% had more than five lesions at first diagnosis.

All patients underwent baseline computed tomography (CT) and/or magnetic resonance imaging (MRI). Nuclear imaging modalities included: 99mTc-HYNIC-TOC (Tektrotyd) scintigraphy (*n* = 56, 29.2%), 68Ga-DOTA PET/CT performed in Switzerland, Germany, or France (*n* = 17, 8.9%), and 18F-fluorodeoxyglucose (FDG) PET/CT (*n* = 13, 6.8%), including 8 of the 19 patients with NEC. Overall, 59 patients (30.7%) underwent somatostatin receptor (SSTR) imaging at least once.

### 3.2. Systemic Treatment

Ninety-eight patients had metastatic disease at diagnosis, and an additional 32 patients developed metastases during surveillance. A total of 131 patients (68.2%) received conventional systemic therapy (excluding PRRT). The most common first-line systemic therapies were SSA (60.3%) and chemotherapy (36.6%). The treatment sequence is detailed in Supplementary Figure S2. Only 39.2% of the patients treated with SSA (31/79) underwent any SSTR imaging. Seventy-nine patients had an indication for first-line SSA and had a median time to treatment initiation of 78 days IQR (42.7–130.2) (*n* = 36; exclusion criteria: SSA initiated before presentation *n* = 24; SSA initiated for recurrence *n* = 18; missing data *n* = 1; sensitivity range 67–72). Of 31 patients with carcinoid syndrome, 19 were operated upfront with curative intent, and 12 received SSA after a median time to treatment initiation of 60.5 days, IQR (4.5–111.3). Thirteen of 19 patients with NEC received first-line chemotherapy, with a median time to treatment initiation of 29 days (IQR (12–45); *n* = 3 unfit for treatment, *n* = 3 operated upfront with curative intent).

### 3.3. Surgery

Overall, 146 surgical procedures were performed on 133 patients (69.3%), including curative (*n* = 103, 70.5%), cytoreductive (*n* = 22, 15.1%), and palliative-intent (*n* = 21, 14.4%) surgeries. Pancreatic resections and enterectomies were the most common surgical procedures. Most surgeries were performed upfront as the first therapeutic intervention, without prior treatment (86.9%), and were carried out at our institution (66.4%).

Ninety-five patients (49.5%) were operated on with curative intent, of whom 15 (15.8%) had metastases. The rate of complete resection (R0) was 98.9%, and 60% (57/95) remained disease-free during a median follow-up of 43 months, IQR (12–94), after surgery. Recurrence was documented in 38 patients (40%), most often new distant metastases (*n* = 30), and a median disease-free interval (DFI) of 19 months, IQR (12.5–48) (*n* = 35 with available data). An overview of surgical management and outcomes is provided in Supplementary Tables S1 and S2.

Postoperative complications were recorded in 17/133 operated patients (12.8%). Pancreatic fistula (*n* = 6), hemorrhagic events (*n* = 5), and infection (*n* = 5) were the most frequent. Most complications were minor (*n* = 11, 8.3%) and resolved with conservative management. One postoperative death following sepsis and multiple organ failure was reported (Supplementary Table S3).

To assess time to surgery, only surgeries performed upfront at our institution were considered (*n* = 82). Emergency procedures and surgeries performed in patients referred specifically for surgical treatment (*n* = 40) occurred with a median of 3 days, IQR (1–4), as their first presentation coincided with the immediate preoperative setting. Among the remaining 42 procedures, the median time from initial presentation to surgery was 30 days, IQR (14.2–71).

The median hospital stay for surgeries performed in our institution was 9 days, IQR (7–14), varying with the complexity of the procedure. Pancreatic resections generally had longer stays (20 days, IQR (12–30.5) for pancreatoduodenectomy and 9 days, IQR (7.5–14) for distal pancreatectomy), while enterectomies or metastasectomies had a median of 7 days of hospitalization (IQR (6–8.3) and (7–11.5), respectively).

### 3.4. Minimally Invasive Treatment

Among 118 patients with synchronous and metachronous liver metastases, 16 (13.6%) underwent 18 liver-directed therapies (LDTs) performed in-house. The procedures consisted of 11 transarterial embolizations (TAEs) with or without an active agent, 3 selective internal radiation therapies (SIRTs), and 4 radiofrequency ablations (RFAs). One additional patient with pancreatic insulinoma underwent RFA for the primary tumor and obtained complete remission during follow-up. Multiple sessions of the same technique were counted as a single intervention.

The LDT group had a balanced distribution between sexes, with a median age of 65 years, IQR (56–67). Most patients originated from the capital city or other urban areas (*n* = 13). Most tumors were grade 2 (*n* = 12), originating in the pancreas (*n* = 11) or small bowel (*n* = 4). Six patients had functioning tumors, mostly carcinoid syndrome (*n* = 4). Most LDTs were performed with palliative intent (*n* = 13). Ten patients had progressive disease after LDT at a median follow-up of 20 months, IQR (5.3–63.8). No statistically significant difference was observed between rural and urban areas (*p* = 0.290), or with age at a 65-year cutoff (*p* = 1.000).

Locoregional therapies were generally integrated early in the treatment sequence, in the first two treatment lines (Supplementary Material Figure S2). The median time from treatment indication to procedure was 62.5 days (IQR (42.3–123.3), *n* = 16/18 available data, sensitivity range 51–84).

Procedure-related complications were reported exclusively after TAE (*n* = 8). The most common adverse events were post-embolization syndrome (*n* = 3) and access-site hematoma (*n* = 2). Single cases of bilioma, liver abscess, and asymptomatic splenic infarction concomitant with mild acute pancreatitis were reported. All patients recovered completely with monitoring or symptomatic treatment. The patient with liver abscess had a history of pancreaticoduodenectomy as a risk factor and required percutaneous drainage. No treatment-related deaths were recorded.

### 3.5. Peptide Receptor Radionuclide Therapy (PRRT)

Among 130 patients with synchronous or metachronous metastases, PRRT was administered to 19 patients (14.6%), of whom 3 patients underwent a second round (re-PRRT). Only the initial PRRT course was considered for further analyses. The male-to-female ratio was 12:7, and the median age at intervention was 59 years, IQR (54–65). Most patients resided in the capital city or other urban areas (*n* = 17). Significant differences were observed by residence (*p* = 0.045), but not by age at a 65-year cutoff (*p* = 0.066). No adjustment for confounding was performed.

The primary tumor sites were: the pancreas (*n* = 7), small bowel (*n* = 3), lung (*n* = 3), unknown primary tumor (*n* = 3), colorectal area (*n* = 2), and appendix (*n* = 1). Most tumors were Grade 2 (*n* = 13), and seven patients had functioning tumors, mostly carcinoid syndrome (*n* = 5). PRRT was also introduced relatively early in the treatment sequence, most often in the 2nd line (Supplementary Material Figure S2).

As PRRT is not available in Romania, all patients were referred to centers abroad, likely contributing to a median time to procedure of 144 days (IQR (91–224), *n* = 14/19 available data, sensitivity range 103–186). One patient received PRRT with 90Y-DOTATOC, and the remaining 18 received 177Lu-DOTATATE; 9/19 patients received fewer than four cycles. Treatment-related toxicity was reported in three patients. Adverse events included carcinoid crisis after the fourth cycle, which resolved with medical treatment; one patient with reversible grade 2 thrombocytopenia and persistent grade 3 chronic kidney disease following re-PRRT; and another case of persistent grade 2 pancytopenia. No treatment-related deaths were recorded.

**Figure 2:**
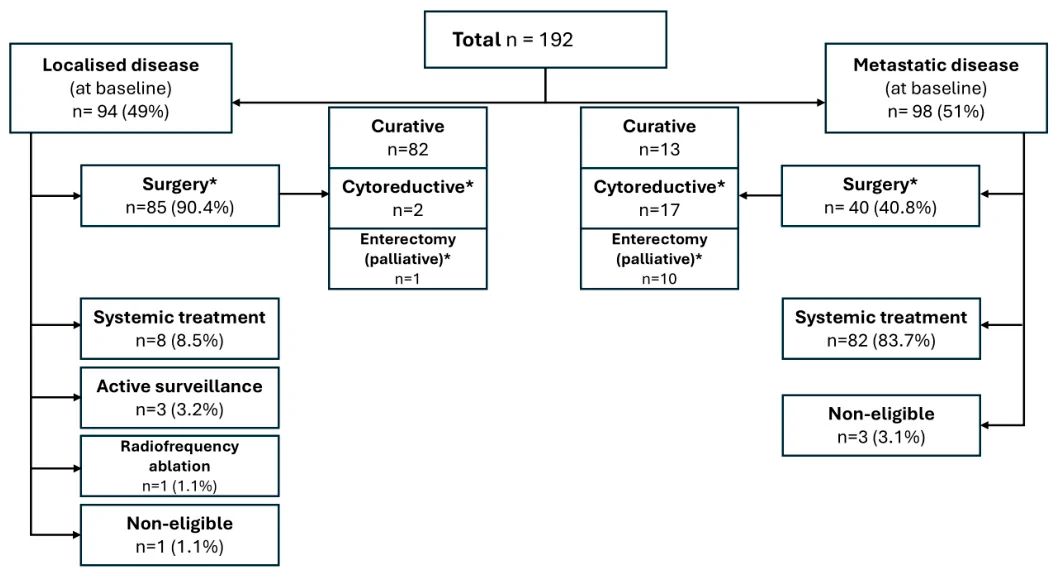
Initial treatment strategies according to disease status at baseline. Treatment categories marked with an asterisk (*) were not mutually exclusive.

**Figure 3:**
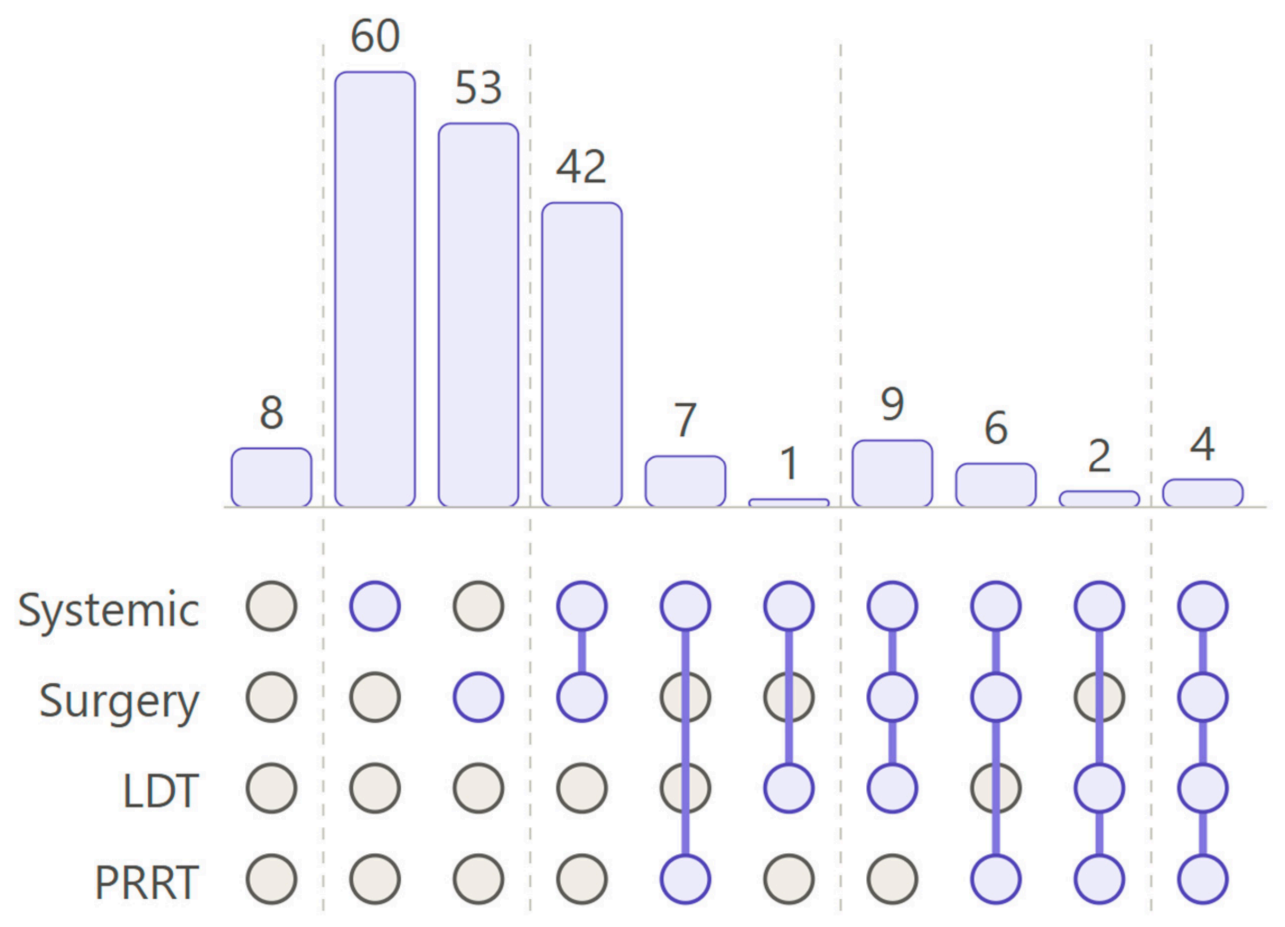
Treatment patterns and combinations of systemic therapy, surgery, liver-directed therapy (LDT), and peptide receptor radionuclide therapy (PRRT) in the study cohort. Bars: total number of patients receiving each treatment combination; purple circles: treatments received; gray circles: treatments not received. Connecting lines link treatment modalities administered to the same patients.

The first treatment options in localized and metastatic disease, respectively, at baseline are detailed in Figure 2. Over the course of the disease, 79 patients (41.1%) received multimodal treatment, including 21 (10.9%) who received at least three treatment modalities (Figure 3).

## 4. Discussion

Our study shows real-world management of neuroendocrine neoplasms in a single tertiary referral center from Eastern Europe and is one of the few comprehensive overviews of clinical practice in our country. We identified 192 patients with NEN treated in our institution between 2011 and January 2026. The annual number of newly diagnosed patients increased approximately fourfold after 2016 with a modest decline during the COVID-19 pandemic, in line with the global trends [8].

In several Eastern European countries, including ours, 99mTc-HYNIC-TOC (Tektrotyd^®^, POLATOM, Otwock, Poland) is the only available SSTR imaging agent [12,13]. While 68Ga-DOTA-peptide PET/CT is the gold standard imaging for well-differentiated NENs [14], 99mTc-HYNIC-TOC is a practical, cost-effective alternative, with a lower radiation burden and higher sensitivity than 111In-pentetreotide scintigraphy (Octreoscan^®^, Curium US LLC., Maryland Heights, MO, USA) [12,15].

Less than one-third of our cohort, and 39.2% of patients receiving SSA monotherapy, underwent SSTR imaging, slightly lower than reported in the published literature (Table 2). Although not mandatory, SSTR assessment is recommended before initiating SSA, given the heterogeneous and potentially discordant SSTR expression compared to immunohistochemistry [3]. An ENETS Center of Excellence reported that 27% of patients referred from other centers had undergone SSTR imaging before referral [3], while discordant findings between SSTR imaging and immunohistochemistry are around 24% [16].

68Ga-DOTA PET/CT was performed abroad in 8.9% of patients, mainly to confirm SSTR expression before PRRT. Reported use of SSTR imaging varies considerably, reaching up to 100% in Centers of Excellence [3,17,18], predominantly by 68Ga-DOTA PET/CT, compared with 27% to over 60% in real-world cohorts from less specialized settings [3,13,19].

Notably, in our cohort, the findings were largely concordant between 99mTc-HYNIC-TOC and 68Ga-DOTA PET/CT, except for one case where 68Ga-DOTA-PET/CT identified additional metastases in the heart and pancreas. Despite the limited availability of advanced SSTR imaging, the proportion of occult primary tumors in our cohort (8.3%) was within the range reported in the literature. NENs are known to have a higher rate of unknown primary tumors, up to 13% [20].

The use of FDG-PET/CT was more consistent across reported cohorts, and our rate is comparable to the range of 25–31% reported in the literature for NEC [13,21,22]. 18F-fluorodopa PET, available only in selected expert centers, was not performed in any patient, as it is not available locally and its role is considered limited by recent guidelines [23].

We analyzed the time to treatment initiation in patients with metastatic carcinoid syndrome and NEC, since these two scenarios carry a particular urgency. We found a median interval of 2 months in carcinoid syndrome, comparable to the reported literature [24]. ENETS recommends prompt treatment in this setting without defining a specific time-to-treatment target [25]. In NEC patients, chemotherapy was initiated after a median of 29 days from first presentation in our center. We consider a one-month interval to be a reasonable target [2,22]. Median survival in NEC was 18 months, which is in line with published reports [26].

In parts of Eastern Europe, access to PRRT is dependent on foreign centers [27]. Although legislation may allow reimbursement for treatment performed abroad, the process often requires prior authorization and, in many cases, upfront payment by the patient, creating additional barriers to care.

We identified 19 patients who received PRRT, of whom 3 had additional cycles. While Centers of Excellence and other high-volume institutions report dozens to hundreds of PRRTs performed in NENs [28], data from countries where PRRT is not available locally consist largely of case series and case reports (Table 2). One study compared the use of PRRT in stage IV pancreatic NEN treated at two Canadian cancer centers; PRRT was performed in 60% of the patients at the center with local PRRT access versus only 6% at the non-PRRT center [29].

In our cohort, the median waiting time to PRRT was 4.8 months. Published data on time to PRRT start is scarce and difficult to compare because reference points differ and availability has evolved over time. Delays exceeding 3 months have been reported in settings with limited availability or reimbursement constraints [30], while even established PRRT centers have reported mean intervals exceeding 6 weeks up to 3 months [30,31].

It is possible that more patients could have benefited from PRRT, although the number is difficult to estimate. Among 26 patients who received at least 3 lines of systemic therapy (excluding surgery and PRRT), only 5 underwent PRRT. Likewise, among the 13 patients with midgut NENs who progressed on somatostatin analogs, only 2 received PRRT. In the remaining cases, second-line treatment consisted mostly of everolimus, followed by capecitabine/oxaliplatin (CAPOX)/5-flurouracil/oxaliplatin (FOLFOX) regimens. Eligibility for PRRT was not assessed retrospectively in untreated patients; therefore, these observations are insufficient to demonstrate PRRT underutilization.

Access to locoregional therapies is also uneven, as they require expertise and dedicated infrastructure. At our institution, liver embolizations and radiofrequency ablations are performed in-house, most commonly for hepatocellular carcinoma.

We identified 16 patients who underwent 18 LDTs (13.6%), which is consistent with published real-world cohorts [13,32], although rates approaching one-third have been reported in specialized centers [33,34] (Table 2). The safety profile was generally favorable, although recurrence and progression were common. This might reflect the pattern of liver disease in our cohort, with multiple, small metastases occupying a relatively limited proportion of both liver lobes. Median time to procedure was approximately 2 months. Although it can be performed locally, time to SIRT was considerably longer, as it became available nationally only in early 2025 and is not yet reimbursed by the health insurance system.

Ablation of the primary tumor (e.g., EUS-guided radiofrequency ablation), an emerging technique used mainly for functioning pancreatic NETs [35], is not routinely performed at our institution; we identified a single patient with insulinoma who underwent this procedure, achieving complete remission.

Despite a relatively balanced geographic distribution within the cohort and similar disease stage at diagnosis across residence subgroups, only two patients from rural areas received PRRT (*p* = 0.045) and three underwent liver-directed therapy (*p* > 0.05), raising concerns about potential geographic barriers to accessing advanced treatments (these findings are hypothesis-generating rather than demonstrative). The median age at the time of PRRT was 59 years, lower than that reported in the NETTER-1 trial [36] and in major real-world series [37]. While younger patients may be more willing to accept the logistical burden that comes with international care, the difference was not statistically significant (*p* = 0.066).

Given the limited availability of locoregional therapies and PRRT, many Eastern European centers rely more on surgery and systemic treatment. In our cohort, 133 patients (69.3%) underwent surgery, most often upfront and with curative intent, resulting in a high rate of R0 resections. These findings are consistent with current guidelines that recommend early complete resection whenever feasible [38].

While some procedures were performed in external hospitals, mostly small-bowel resections, approximately two-thirds of all surgeries were carried out at our institution, which is a referral center for hepato-pancreatico-biliary surgery. As a result, the surgical portfolio included more complex procedures, predominantly pancreatic resections. Postoperative morbidity and mortality were within the expected range [39], with an acceptable hospital stay despite the limited use of laparoscopic surgery, performed in only one case. The 1-month interval from first presentation to surgery was also acceptable. Longer intervals have been reported in some high-volume centers compared with smaller centers [2,40,41]. However, spleen preservation was achieved in only one-third of the tumors located in the pancreatic tail, lower than the rates reported by Centers of Excellence [2].

A recurrence rate of 40% in a predominantly R0-resected cohort may raise concerns, particularly given that the median follow-up of 43 months may underestimate long-term relapses, which are common in this disease. The literature data vary substantially according to patient selection criteria and follow-up, but most studies that included mixed NEN populations reported recurrence in approximately one quarter to one third of patients [42]. More optimistic results of 10–20% generally come from more selected populations, either with intestinal primaries predominating, or those restricted to non-metastatic disease [43]. Taken together, the observed recurrence rate is consistent with the published literature.

**Table 2:** Comparison of selected diagnostic and treatment parameters between the present cohort and published cohorts from non-specialized centers and high-volume/ENETS Centers of Excellence.

Parameter	Original Cohort	Non-Specialized Centers	ENETS Center of Excellence/ Specialized High-Volume Centers
SSTR imaging	99mTc-HYNIC-TOC: 29.2% (56/192); 68Ga-DOTA PET: 8.9% (17/192).	Santos et al. [32], 2019: Octreoscan: 41.3% (121/293 GEP-NEN); 68Ga-DOTA PET: 33.8% (99/293 GEP-NEN).	Slott et al. [18], 2024: 100% (615/615 siNEN), Octreoscan before 2012, 64Cu/68Ga-DOTA PET after 2012. Mathew et al. [17], 2023: 68Ga-DOTA PET: 100% (314/314 pNEN).
PRRT	14.6% (19/130 metastatic NEN).	Nair et al. [29], 2025: 6.3% (7/112 pNET); Alfagih et al. [44], 2023: 4% (7/177 siNET).	Melhorn et al., 2022 [45]: 54.5% (42/77 metastatic siNET). Nair et al. [29], 2025: 59.7% (46/77 pNET)
Liver-directed therapy (LDT)	13.6% (16/118 NEN with liver metastases).	Santos et al. [32], 2019: 14.7% (14/95 metastatic NEN); Nikou et al. [13], 2016: 14.3% (7/49 NEN with liver metastases).	Fairweather et al. [33], 2017: 649 NEN with liver metastases RFA 4.3% (28/649); TACE 20% (130/649). Clift et al. [34], 2023, 154 siNET: SIRT 20.1% (31/154); RFA 6.5% (10/154); TACE 1.3% (2/154).
R0 resection	98.9% (94/95 NEN resected with curative intent)	Søreide et al. [46], 2022: 84% (126/150 GEP-NEN surgical resections).	Morin et al. [2], 2022: 100% (13/13 pNEN; 92.6% (25/27 siNEN).

Abbreviations: GEP-NEN, gastroenteropancreatic neuroendocrine neoplasm; LDT, liver-directed therapy; NEN, neuroendocrine neoplasm; pNET/pNEN, pancreatic neuroendocrine tumor/neoplasm; PRRT, peptide receptor radionuclide therapy; RFA, radiofrequency ablation; siNET/siNEN, small-intestinal neuroendocrine tumor/neoplasm; SIRT, selective internal radiation therapy; SSTR, somatostatin receptor; TACE, transarterial chemoembolization. Note: Results are presented as % (n/denominator). For LDT studies reporting multiple modalities, modalities were not summed because overlap between treatment groups was not specified.

This study is limited by its retrospective, single-center design, descriptive analysis, absence of central pathology review, and relatively short median follow-up (5 years) given the natural history of NETs. As inclusion criteria required at least one treatment, the cohort likely over-represents patients with greater disease complexity and findings may therefore not be representative of the broader NEN population. Additionally, using first presentation to our center as a reference point for time-to-treatment intervals excludes any pre-referral waiting time. Eligibility for PRRT and locoregional therapies could not be retrospectively assessed, limiting our ability to draw firm conclusions on treatment access or identify potentially vulnerable patient populations. The absence of a dedicated ICD-10 code (International Classification of Diseases, 10th Revision) for NENs may have resulted in incomplete case ascertainment, despite extensive manual screening. PFS and further stratification of outcomes by primary site or grade were not assessed due to the small cohort size and heterogeneity in treatment approaches. These findings describe practice at a single tertiary referral center operating within a specific national healthcare and reimbursement framework and may not be generalizable to other Eastern European centers.

## 5. Conclusions

This is one of the few reports on real-world management of NENs in an Eastern European referral center. Although multidisciplinary care is available, the use of advanced SSTR imaging, liver-directed therapies, and PRRT was within the range reported in centers without dedicated NEN expertise, but lower than rates described in high-volume centers.

Our findings highlight several practical areas for improvement: reducing treatment delays, improving access to PRRT and locally available liver-directed therapies, and developing more structured NEN care pathways to facilitate multimodal treatment. Addressing these issues could improve the timeliness and continuity of care and further align practice with that of established Centers of Excellence.

## Figures and Tables

**Table 1:** Patients’ characteristics.

Characteristic	Category	N = 192 (%)
Age at diagnosis (years)	Median, (IQR)	60 (50–67)
Sex	Male	89 (46.4%)
	Female	103 (53.6%)
Area of residence	Capital metropolitan	60 (31.3%)
	Other urban	67 (34.9%)
	Rural	65 (33.9%)
Primary site *	Pancreas	115 (59.9%)
	Small bowel	43 (22.4%)
	Unknown primary	16 (8.3%)
	Lung	6 (3.1%)
	Stomach	4 (2.1%)
	Colon	3 (1.6%)
	Rectum	3 (1.6%)
	Other	3 (1.6%)
Grade	G1	81 (42.2%)
	G2	88 (45.8%)
	G3—well-differentiated	3 (1.6%)
	G3—poorly differentiated	19 (9.9%)
	Not reported	1 (0.5%)
Functional status	Non-functioning	148 (77.1%)
	Carcinoid syndrome	31 (16.1%)
	Insulinoma	6 (3.1%)
	Gastrinoma	3 (1.6%)
	Other **	4 (2.1%)
Stage at diagnosis	Non-metastatic	94 (49%)
	Metastatic	98 (51%)
Number of metastatic sites at diagnosis	1 site	56 (29.2%)
	2–3 sites	34 (17.7%)
	4–7 sites	8 (4.2%)

* One patient had 2 primary NENs in the pancreas and ileum. ** Other functioning tumors included: Glucagonoma (*n* = 2), Cushing syndrome (*n* = 1), Serotoninoma (*n* = 1).

## Data Availability

The data presented in this study are available on request from the corresponding author. Data are not publicly available due to patient privacy and institutional data protection policies.

## Supplementary Materials

The following supporting information can be downloaded at: https://www.mdpi.com/article/10.3390/jcm15197762/s1, Figure S1: Flowchart of patient selection; Figure S2. Sequencing of systemic and liver-directed therapies in the study cohort. Patients may have undergone multiple LDT and/or PRRT procedures. LDT, liver-directed therapy; PRRT, peptide receptor radionuclide therapy; Table S1: Types of surgery. Categories are not mutually exclusive; Table S2. Outcomes in patients undergoing curative-intent surgery; Table S3: Post-operative morbidity and mortality based on Clavien–Dindo classification. * Multiple categories may apply to the same patient.

## Abbreviations

The following abbreviations are used in this manuscript:

DFI	Disease-free interval
ECOG	Eastern Cooperative Oncology Group performance status
ENETS	European Neuroendocrine Tumor Society
MDT	Multidisciplinary tumor board
MEN	Multiple endocrine neoplasia
NEC	Neuroendocrine carcinoma
OS	Overall survival
PRRT	Peptide receptor radionuclide therapy
RFA	Radiofrequency ablation
SIRT	Selective internal radiation therapy
SSAs	Somatostatin analogs
SSTR	Somatostatin receptor
TAE	Transarterial embolization
VHL	Von Hippel–Lindau syndrome
