# Supplementary material for: Multidisciplinary Management of Neuroendocrine Neoplasms Beyond Centers of Excellence: Retrospective Real-World Data from a Referral Center in Eastern Europe
Source: J Clin Med. 2026 Oct 8;15(19):7762. doi: 10.3390/jcm15197762 (PMC13666263; doi:10.3390/jcm15197762)
Supplement: Supplementary file 1 [file jcm-15-07762-s001.zip › jcm-4574056-supplementary.pdf]

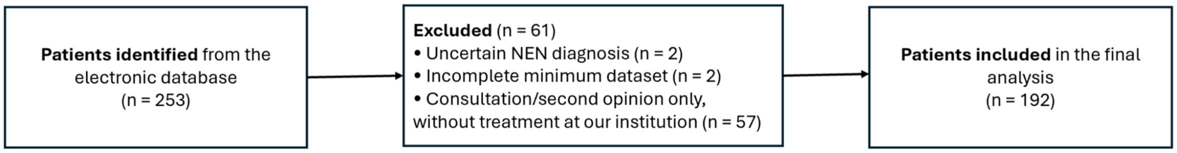


**Figure S1.** Flowchart of patient selection

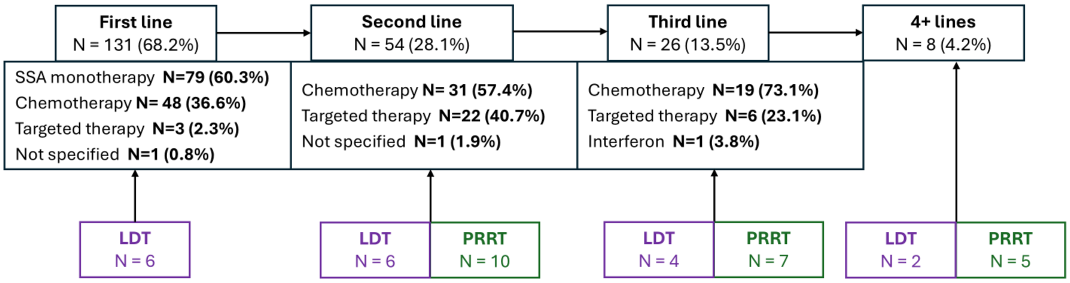


**Figure S2.** Sequencing of systemic and liver-directed therapies in the study cohort. Patients may have undergone multiple LDT and/or PRRT procedures. LDT, liver-directed therapy; PRRT, peptide receptor radionuclide therapy.

| **Type of surgery** | **N=146 (%)** |
|---|---|
| Enterectomy | **N=36 (24.7%)** |
| Pancreatic resection | **N=70 (48.0%)** |
| Pancreatosplenectomy **N=34** | |
| Spleen-Preserving Distal Pancreatectomy **N=18** | |
| Duodenopancreatectomy **N=13** | |
| Enucleation **N=4** | |
| Central pancreatectomy **N=1** | |
| Hemicolectomy | **N=5 (3.4%)** |
| Gastrectomy | **N=4 (2.7%)** |
| Liver resection with primary tumor | **N=13 (8.9%)** |
| Liver resection without primary tumor | **N=17 (11.6%)** |
| Other | **N=14 (9.6%)** |

**Table S1.** Types of surgery. Categories are not mutually exclusive.

| Characteristic | Category | n (%) |
|---|---|---|
| Stage | Localized | 80 (84.2%) |
| | Metastatic | 15 (15.8%) |
| Type of resection | R0 | 94 (98.9%) |
| | R1 | 1 (1.1%) |
| Outcome | Complete remission | 57 (60%) |
| | Recurrence | 38 (40%) |
| Type of recurrence | Distant metastases | 30/38 (78.9%) |
| | Local recurrence | 6/38 (15.8%) |
| | Combined | 1/38 (2.6%) |
| | Missing data | 1/38 (2.6%) |

**Table S2.** Outcomes in patients undergoing curative-intent surgery.

| Complication* | Clavien–Dindo grade, n |
|---|---|
| Fistula (after pancreatic resection) | Grade I-II: n= 3;<br>Grade III: n= 3 |
| Hemorrhagic complication | Grade I-II: n= 2;<br>Grade III: n= 3 |
| Infectious complication | Grade I-II: n= 2;<br>Grade III: n= 3 |
| Emetic syndrome (delayed oral feeding) | Grade I-II: n= 2 |
| Pulmonary embolism | Grade II: n=1 |
| Pancreatitis | Grade II: n=1 |
| Hydroureteronephrosis | Grade III: n=1 |
| Postoperative collection requiring drainage | Grade III: n=1 |
| Death | Grade V: n= 1 |

**Table S3.** Post-operative morbidity and mortality based on Clavien–Dindo classification. * Multiple categories may apply to the same patient.
